# Supplementary figures and images for: scFv intrabody targeting wildtype TDP-43 presents protective effects in a cellular model of TDP-43 proteinopathy
Source: PLoS One. 2025 Aug 4;20(8):e0322021. doi: 10.1371/journal.pone.0322021 (PMC12321133; doi:10.1371/journal.pone.0322021)

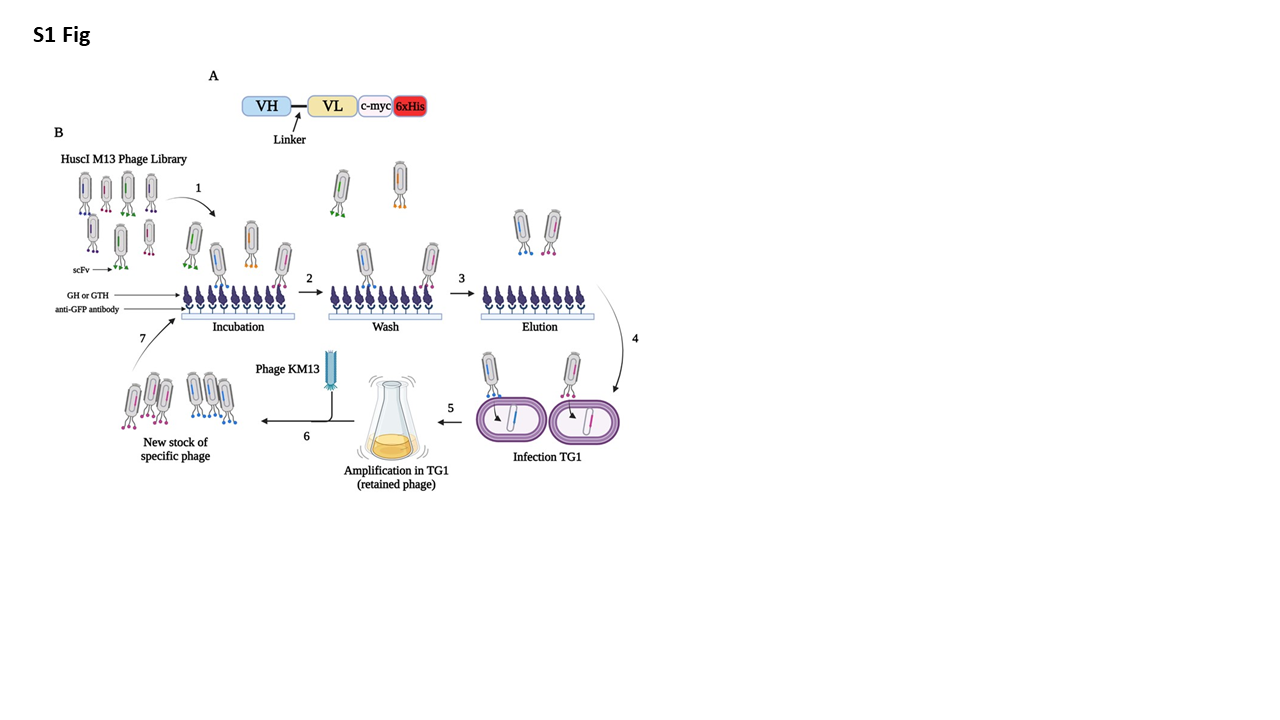

Supplement: S1 Fig — A) General structure of scFv protein. Once expressed as protein isolated from phage, it contains C-terminal c-myc and 6xHis tags. B) The HuscI library contained M13 phage that expressed copies of one scFv at their tail. The library that was depleted against anti-GFP antibody and against GFP-6xHis (GH) was incubated with GFP-wtTDP-43-6xHis (GTH) immobilized on anti-GFP antibody (1). After washing (2), the adhered phage were eluted by trypsin (3) and induced to infect TG1 (4). Following amplification of infected TG1 (5), new phage particles were produced (6) containing amplified specific phage. These phage were re-incubated with GTH (7). The cycle was repeated a total of 5 times. Figure made with Biorender.com. (TIF) [file pone.0322021.s001.tif]

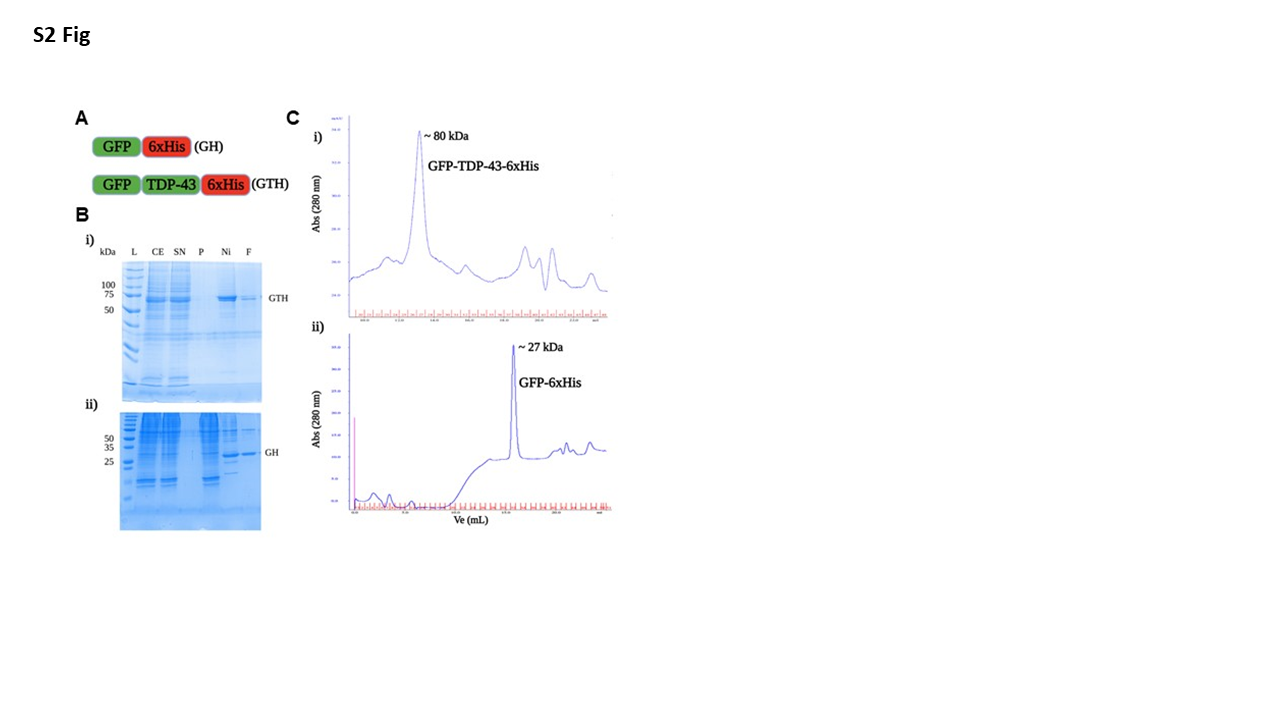

Supplement: S2 Fig — A) Structures of the recombinant proteins GFP-6xHis (GH) and GFP-TDP-43-6xHis (GTH) of 28 kDa and 75 kDa, respectively. B) SDS-PAGE gel stained by Coomassie reagent representing the purification of i) GTH and ii) GH. L: ladder; CE: crude extract of lysate. SN: supernatant of lysate. P: pellet of lysate. Ni: pooled eluted protein from affinity chromatography. F: final product following dialysis. C) Gel filtration profile of dialyzed i) GTH and ii) GH. The chromatogram reflects highly pure monomers of each protein. Ve: elution volume. (TIF) [file pone.0322021.s002.tif]

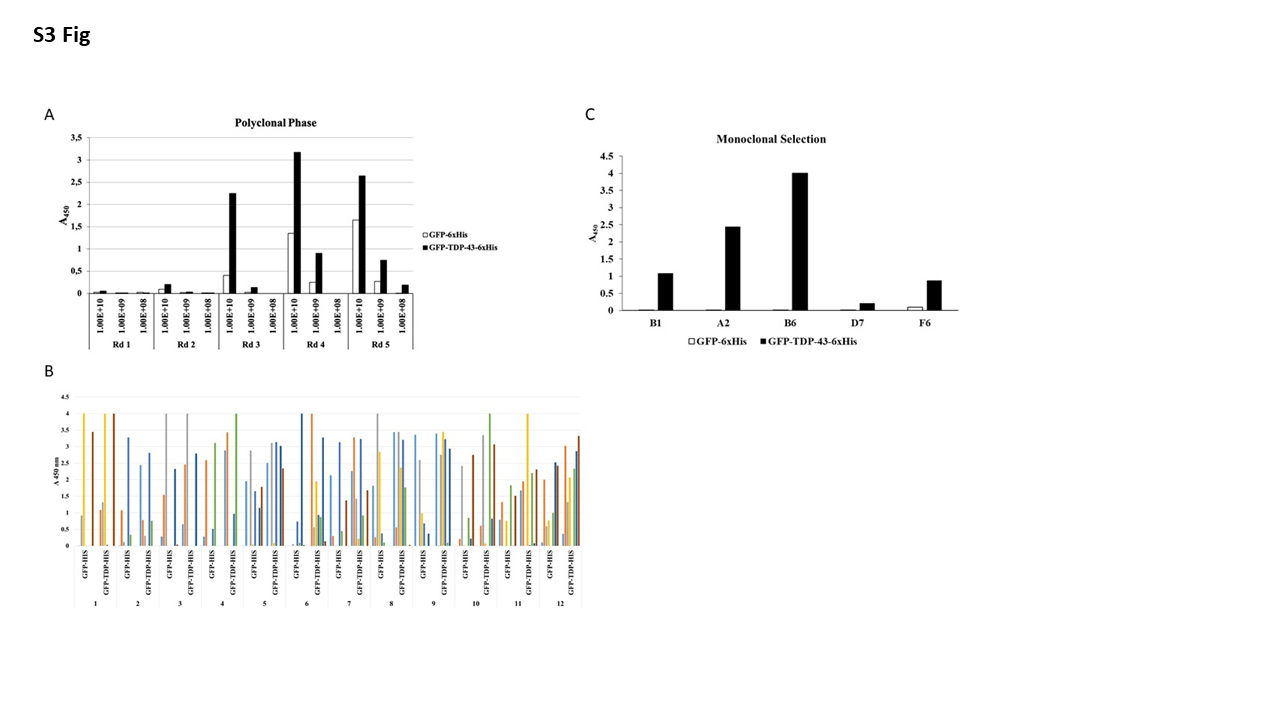

Supplement: S3 Fig — Each round of eluted phage was tested by ELISA on wells coated with GH and GTH antigen. The x-axis represents the 3 dilutions in phage/mL used on coated wells. The white bar represents the absorbance at 450 nm for GH incubation, while the black bar represents the absorbance for GTH incubation. Round 4 appears to contain the most phage with affinity for GTH. B) Monoclonal screen of selected phage from round 4 for binding to GH and GTH. C) Identification of monoclonal anti-TDP-43 scFv-phage display from TG1. Phage with distinct scFv sequences from wells B1, A2, B6, and D7 were identified as anti-TDP-43 because no absorbance was detected with GH. Phage from well F6 is represented as an example of non-specific binding because absorbance was also detected when incubated with the GH control. (TIF) [file pone.0322021.s003.tif]

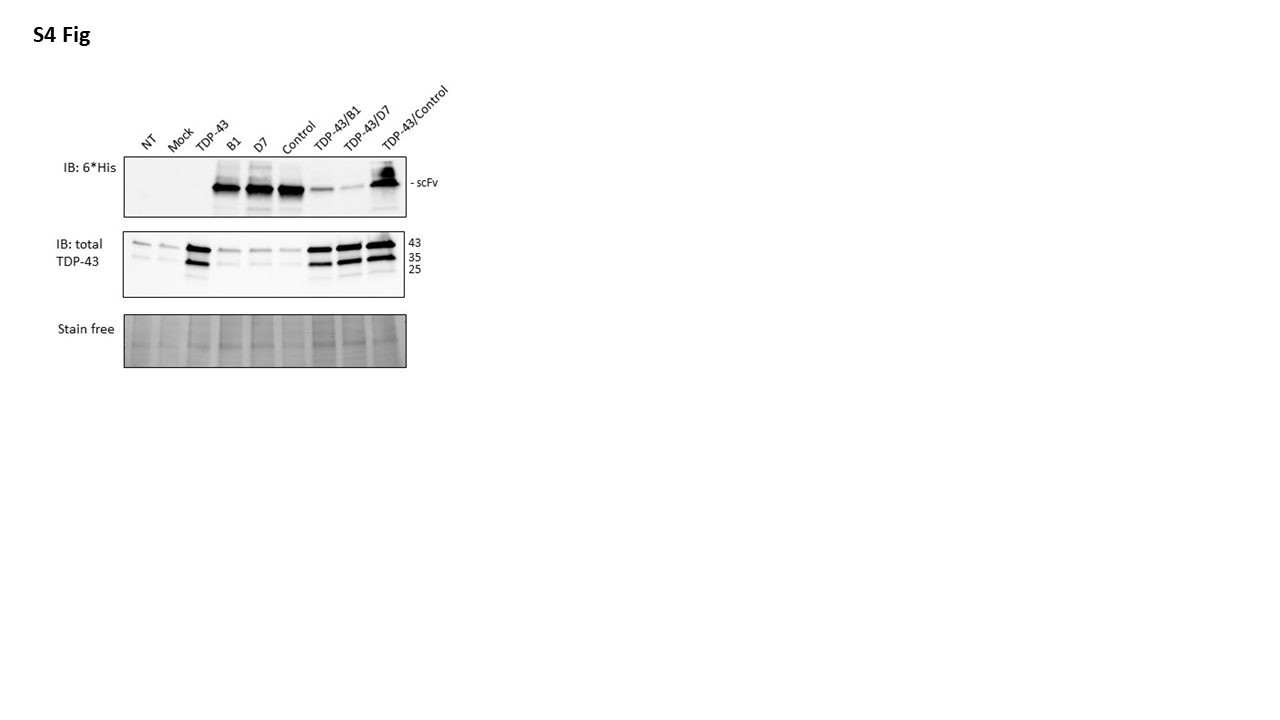

Supplement: S4 Fig — Immunoblots showing the expression of the intrabody when expressed alone and when co-overexpressed with TDP-43. NT: non-transfected. Mock: cells transfected with the empty vector. TDP-43: cells transfected with TDP-43-expressing plasmid and empty vector. B1, D7, control: cells transfected with intrabody-expressing plasmid and empty vector. TDP-43/B1, TDP-43/D7 and TDP-43/control: cells transfected with TDP-43-expressing plasmid and intrabody-expressing plasmid. (TIF) [file pone.0322021.s004.tif]

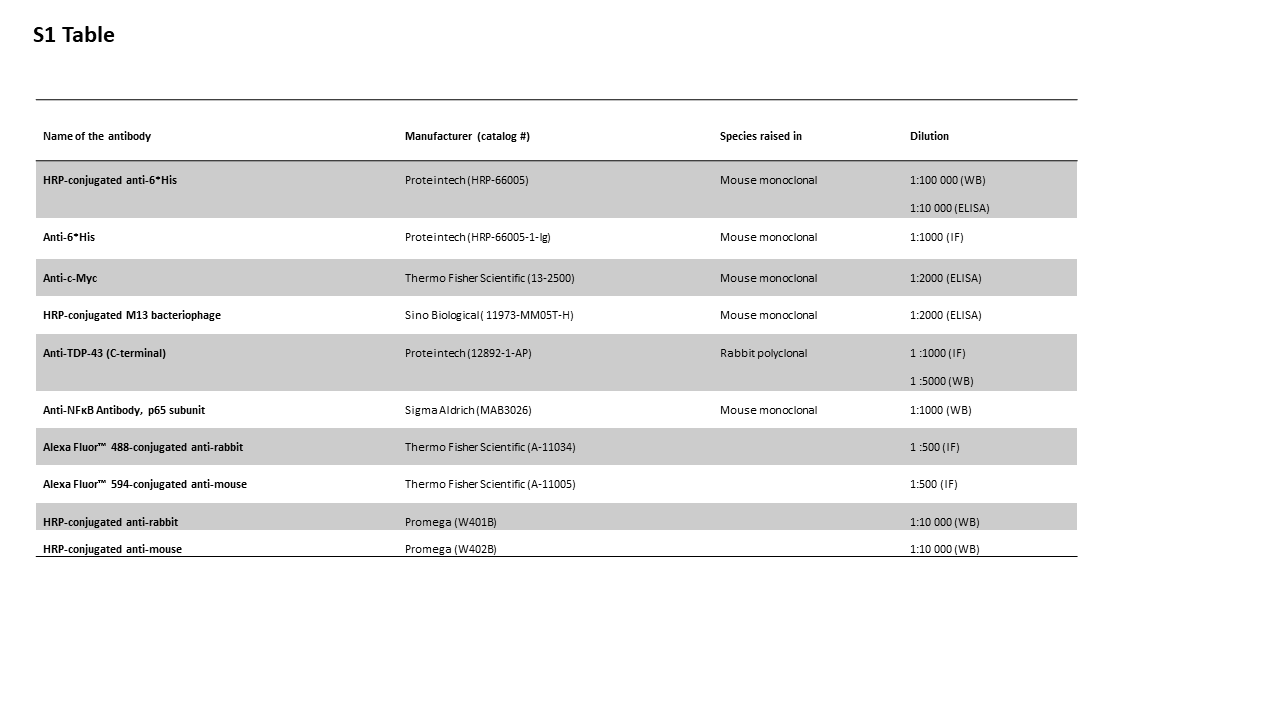

Supplement: S1 Table — (TIF) [file pone.0322021.s005.tif]

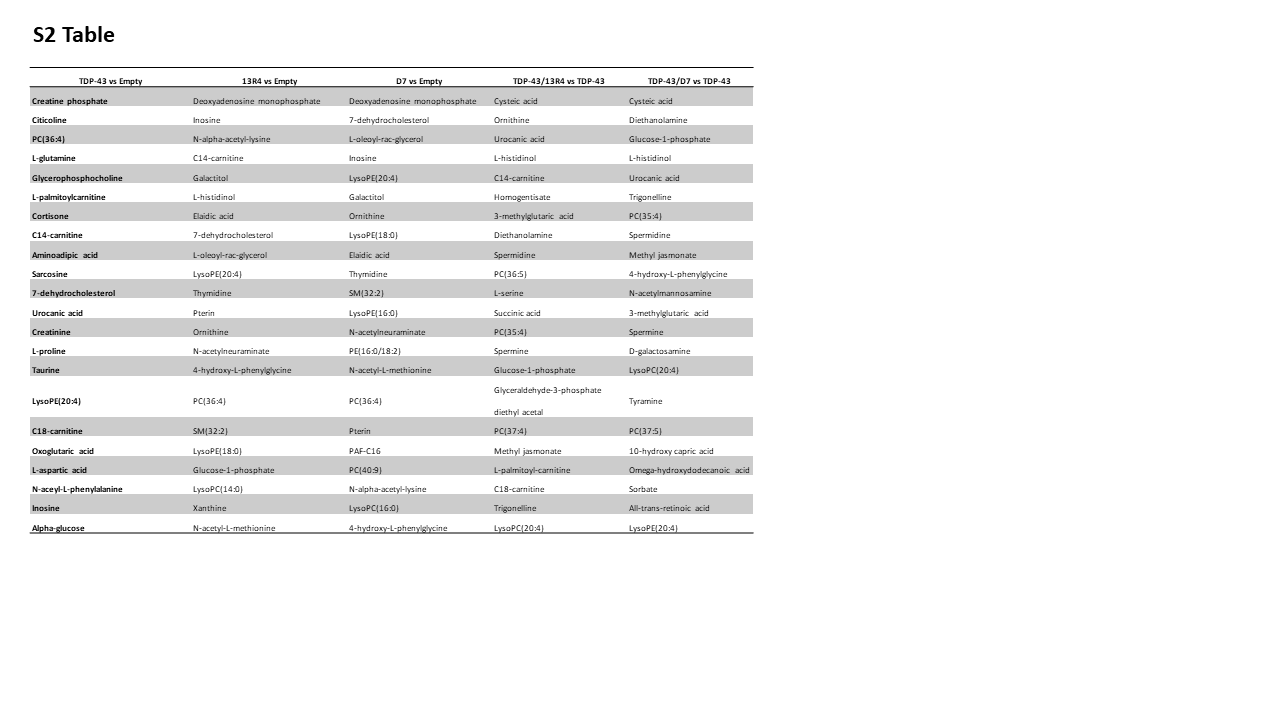

Supplement: S2 Table — (TIF) [file pone.0322021.s006.tif]

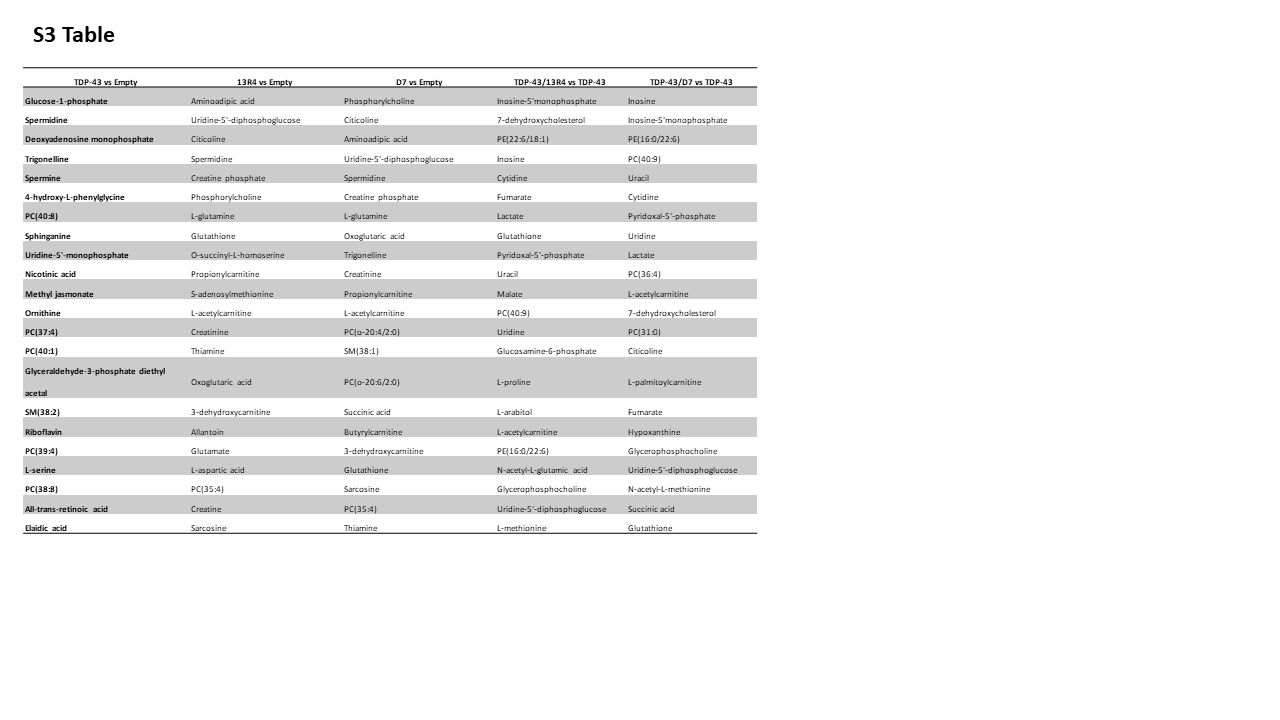

Supplement: S3 Table — (TIF) [file pone.0322021.s007.tif]
